# Combined Treatment with Amlodipine and Atorvastatin Calcium Reduces Circulating Levels of Intercellular Adhesion Molecule-1 and Tumor Necrosis Factor-α in Hypertensive Patients with Prediabetes

**DOI:** 10.3389/fnagi.2016.00206

**Published:** 2016-08-25

**Authors:** Zhouqing Huang, Chen Chen, Sheng Li, Fanqi Kong, Peiren Shan, Weijian Huang

**Affiliations:** The Key Laboratory of Cardiovascular Disease of Wenzhou, Department of Cardiology, The First Affiliated Hospital of WenZhou Medical UniversityWenZhou, ZheJiang, China

**Keywords:** amlodipine and atorvastatin calcium, prediabetes, hypertension, inflammation, endothelial dysfunction, intercellular adhesion molecule-1, tumor necrosis factor-α

## Abstract

**Objective**: To assess the effect of amlodipine and atorvastatin on intercellular adhesion molecule (ICAM)-1 and tumor necrosis factor (TNF)-α expression, as endothelial function and inflammation indicators, respectively, in hypertensive patients with and without prediabetes.

**Methods**: Forty-five consecutive patients with hypertension, diagnosed according to JNC7, were divided into two groups based on the presence (HD group, *n* = 23) or absence (H group, *n* = 22) of prediabetes, diagnosed according to 2010 ADA criteria, including impaired glucose tolerance (IGT) and fasting glucose tests. All patients simultaneously underwent 12-week treatment with daily single-pill amlodipine besylate/atorvastatin calcium combination (5/10 mg; Hisun-Pfizer Pharmaceuticals Co. Ltd). Serum isolated before and after treatment from overnight fasting blood samples was analyzed by ELISA.

**Results**: In the HD and H groups after vs. before 12-week amlodipine/atorvastatin treatment, there were significantly (all *P* < 0.01) lower levels of ICAM-1 (3.06 ± 0.34 vs. 4.07 ± 0.70 pg/ml; 3.26 ± 0.32 vs. 3.81 ± 0.60 pg/ml, respectively) and TNF-α (78.71 ± 9.19 vs. 110.94 ± 10.71 pg/ml; 80.95 ± 9.33 vs. 101.79 ± 11.72 pg/ml, respectively), with more pronounced reductions in HD vs. H group (ICAM-1Δ: 1.01 ± 0.80 vs. 0.55 ± 0.64 pg/ml, respectively, *P* = 0.037; TNF-αΔ: 32.23 ± 14.33 vs. 20.84 ± 14.89 pg/ml, respectively, *P* = 0.011), independent of the blood pressure (BP) and cholesterol level reduction.

**Conclusions**: Amlodipine/atorvastatin improved endothelial function and inflammation, as reflected by lower circulating levels of ICAM-1 and TNF-α, more prominently in hypertensives with than without prediabetes. Starting statin treatment before overt diabetes in hypertensives might thus improve cardiovascular outcomes.

## Introduction

Atherosclerosis, the leading cause of death worldwide, is associated with endothelial dysfunction and inflammation (Libby, 2002; Davignon and Ganz, 2004), with hypertension and prediabetes playing crucial pathogenic roles by increasing secretion of adhesion molecules and inflammatory cytokines (Magen et al., 2010; DeFronzo and Abdul-Ghani, 2011). Overall, 25.9–69.3% of individuals have at least one known risk factor for atherosclerosis, including hypertension, diabetes, dyslipidemia, smoking, and obesity (Rosamond et al., 2007). Hypertension, its most common risk factor, accounts for about half of the global atherosclerosis morbidity and mortality (Ezzati et al., 2005), and a sizable proportion of patients with essential hypertension suffer from prediabetes (García-Puig et al., 2006; Gu et al., 2012), with increased blood glucose levels, both fasting and postprandial, even below the threshold levels of diabetes diagnostic criteria, increasing cardiovascular mortality (Levitan et al., 2004). The hemodynamic changes induced by hypertension lead to endothelial dysfunction, with increased serum levels of inflammation factors such as tumor necrosis factor-α (TNF-α), and expression of adhesion molecules including intercellular adhesion molecules-1 (ICAM-1) further enhancing monocyte/macrophage migration into the vascular wall and promoting atherosclerosis and inflammation, which is further aggravated by other risk factors such as prediabetes (Chapman and Sposito, 2008).

Statins competitively inhibit HMG-CoA reductase and exhibit various pleiotropic effects which attenuate inflammation and improve endothelial function as reflected by decreased levels of TNF-α and ICAM-1 independent of blood lipid level reduction (Ascer et al., 2004; Davignon, 2004; Landsberger et al., 2007). The European Society of Cardiology (ESC)/European Atherosclerosis Society (EAS) guidelines for the management of dyslipidemias therefore recommend statin treatment in patients with hypertension and diabetes regardless of basal lipid level to reduce cardiovascular disease (CVD) risks (Catapano et al., 2011). However, it is unknown whether the latter benefit extends to patients with hypertension and prediabetes, for whom there are scant data on the effects of statin plus anti-hypertensive therapy. The present study therefore investigated the effect of a single-pill combination of amlodipine and atorvastatin calcium on the circulating levels of ICAM-1 and TNF-α in patients with hypertension with or without prediabetes.

## Materials and Methods

### Study Population

All 45 study subjects were enrolled from the cardiovascular outpatient clinic between April 2012 and November 2013, and all assessments were completed. Study exclusion criteria were: coronary heart disease; stroke; secondary hypertension; diabetes; liver disease; rheumatological, neoplastic or other endocrine disease; serum creatinine concentration >1.20 mg/dl; severe dyslipidemia (serum low density lipoprotein-cholesterol (LDL-c) ≥160 mg/dl; total cholesterol (Tc) ≥240 mg/dl or triglycerides >200 mg/dl; recent acute illness; neuropathy; or participation in other clinical trials. Overall, age range was 45–67 years, and no subject had been treated for CVD or other risk factors. Twenty-two hypertensive subjects (H group) and 23 hypertensive prediabetic subjects (HD group) were studied. Hypertension was defined as stage 1 hypertension, i.e., systolic blood pressure (SBP) ≥140 mmHg and <160 mmHg, and diastolic blood pressure (DBP) ≥90 mmHg and <100 mmHg (Chobanian et al., 2003). Blood pressure (BP) was measured twice with a standardized mercury manometer after the subject rested for at least 15 min; the mean of two measurements was used for the analysis. Prediabetes was diagnosed according to 2010 ADA criteria including impaired fasting glucose (IFG) or/and impaired glucose tolerance (IGT; American Diabetes Association, 2010) tests.

### Study Design

All eligible subjects underwent blood testing at baseline, after which all were treated daily with a single-pill combination of amlodipine besylate atorvastatin calcium (5/10 mg; Hisun-Pfizer Pharmaceuticals Co. Ltd). All subjects received dietary instructions, and liver function and creatine kinase level were monitored for potential side effects of statins. After 12 weeks of treatment, all subjects underwent blood testing. The study primary outcome was the changes of circulating levels of ICAM-1 and TNF-α after 12 weeks’ treatment compared with before. The study conformed to the guidelines set forth in the Helsinki declaration, and the protocol was approved by the Medical Ethics Committee of the first affiliated hospital of Wenzhou Medical University. All patients provided written informed consent.

### Laboratory Measurements

All laboratory experiments were carried out after an overnight fast. Blood samples were drawn from an antecubital vein and collected in ethylene diaminetetraacetic acid tubes. Blood specimens were centrifuged at 3000 rpm for 10 min, and the serum was separated and stored at −80°C until analysis. Serum levels of fasting glucose (Glu), glycosylated hemoglobin (HbA1c), 2 h-glucose (2 h-Glu), Tc, LDL-c, alanine amino transferase (ALT) and creatinine (Cr) were determined in the laboratory of the First Affiliated Hospital of Wenzhou Medical University. Serum levels of ICAM-1 and TNF-α, serving as indicators of endothelial function and inflammation, respectively were assessed using commercially available enzyme-linked immunoassay kits in accordance with the manufacturer’s instructions (Bio-Swamp and Rapidbio).

### Statistical Analysis

Normal distribution of the data was tested using Kolmogorov–Smirnov test. All the data were normally distributed. Continuous data are presented as mean ± SD, and were compared using paired samples *T*-test and independent samples *T*-test. Bivariate correlation analysis was performed to explore the relation among ICAM-1, TNF-α, BP, Tc and LDL-c. The 0.05 level of probability was set as being statistically significant. All analyses were performed with the SPSS (version 20.0) statistical package.

## Results

### Baseline Characteristics of Study Participants

As shown in Table 1, there were no significant differences in the distributions of clinical and metabolic characteristics of study participants between H and HD groups except for glucose and HbA1c levels.

**Table 1:** **Baseline clinical characteristics by study group**.

	Hypertension alone (H)	Hypertension and prediabetes (HD)
Male/Female	14/8	19/4
Age, years	53.68 ± 5.07	55.09 ± 6.80
BMI, kg/m^2^	24.84 ± 1.97	25.04 ± 2.17
Smoking, (%)	21.20%	22.90%
Alcohol use, (%)	43.50%	42.10%
SBP, mmHg	147.00 ± 5.67	149.74 ± 6.77
DBP, mmHg	92.86 ± 5.86	95.30 ± 8.00
Glu, mmol/L	5.27 ± 0.25	6.03 ± 0.44*
2 h-Glu, mmol/L	6.10 ± 0.90	8.04 ± 1.43*
HbA1c, (%)	5.48 ± 0.26	5.90 ± 0.33*
Tc, mmol/L	5.67 ± 0.41	5.60 ± 0.70
LDL-c, mmol/L	3.45 ± 0.32	3.30 ± 0.52
ALT, U/L	28.50 ± 12.37	28.70 ± 15.18
Cr, μmol/L	63.18 ± 13.95	69.48 ± 11.05

Data are presented as mean ± SD or as percentages. *P < 0.01, compared with baseline H. Otherwise, P-values were not significant.

### The Effect of the Combination Treatment With Amlodipine and Atorvastatin Calcium on BP and Blood Lipid Levels in Both Study Groups

**Figure 1:**
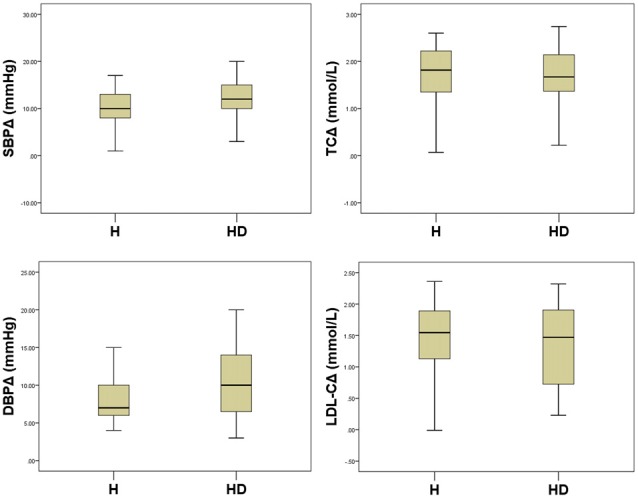
**Comparisons of reductions in Blood pressure (BP) and blood lipid levels between study groups.** All *P* > 0.05.

The 12-week treatment with the single-pill combination of amlodipine 5 mg and atorvastatin calcium 10 mg daily significantly reduced (*P* < 0.01) BP and blood lipid levels in H and HD groups (Table 2) to a statistically similar extent (*P* > 0.05; Figure 1).

**Table 2:** **Clinical parameters by study group before and after treatment**.

	Hypertension (H)		Hypertensive prediabetes (HD)	
	Baseline	12 weeks	Baseline	12 weeks
SBP, mmHg	147.00 ± 5.67	136.77 ± 6.91^#^	149.74 ± 6.77	137.17 ± 4.84^⋇^
DBP, mmHg	92.86 ± 5.86	84.50 ± 5.91^#^	95.30 ± 8.00	84.70 ± 4.64^⋇^
Glu, mmol/L	5.27 ± 0.25	5.25 ± 0.36	6.03 ± 0.44	6.13 ± 0.54
2 h-Glu, mmol/L	6.10 ± 0.90	NA	8.04 ± 1.43	NA
HbA1c, (%)	5.48 ± 0.26	5.45 ± 0.25	5.90 ± 0.33	5.94 ± 0.30
Tc, mmol/L	5.67 ± 0.41	3.98 ± 0.80^#^	5.60 ± 0.70	3.94 ± 0.88^⋇^
LDL-c, mmol/L	3.45 ± 0.32	2.07 ± 0.65^#^	3.30 ± 0.52	1.95 ± 0.54^⋇^
ALT, U/L	28.50 ± 12.37	32.95 ± 15.55	28.70 ± 15.18	39.0 ± 27.50
Cr, μmol/L	63.18 ± 13.95	62.95 ± 14.66	69.48 ± 11.05	67.43 ± 13.67

*Data are presented as mean ± SD. ^#^P < 0.01, compared with baseline H. ^⋇^P < 0.01, compared with baseline HD. NA, not applicable*.

### The Effect of the Single-Pill, Amlodipine and Atorvastatin Calcium Treatment on ICAM-1 and TNF-α Levels in Hypertensive Patients With or Without Prediabetes

**Figure 2:**
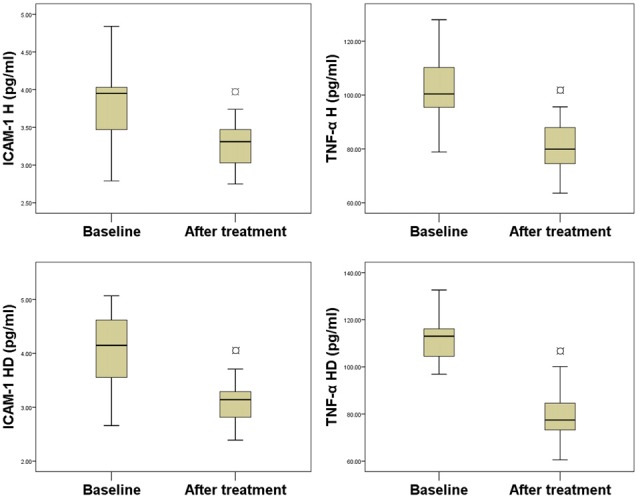
**Comparison between serum levels of biomarkers before and after treatment.** ^¤^*P* < 0.01, compared with baseline.

As shown in Figure 2, after 12 weeks of treatment with the single-pill combination of amlodipine 5 mg and atorvastatin calcium 10 mg daily, ICAM-1 level significantly (*P* < 0.01) decreased by 14.44% in the H group (from 3.81 ± 0.60 pg/ml to 3.26 ± 0.32 pg/ml), and by 24.82% in the HD group (from 4.07 ± 0.70 pg/ml to 3.06 ± 0.34 pg/ml). TNF-α level also significantly (*P* < 0.01) decreased after treatment by 20.47% in the H group (from 101.79 ± 11.72 pg/ml to 80.95 ± 9.33 pg/ml), and by 29.05% in the HD group (from 110.94 ± 10.71 pg/ml to 78.71 ± 9.19 pg/ml).

### The Difference of the Treatment’s Effect on ICAM-1 and TNF-α Levels in Hypertensive Patients With and Without Prediabetes

**Figure 3:**
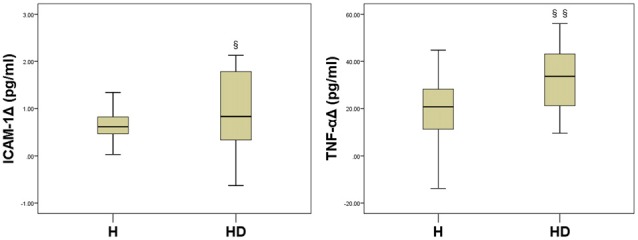
**Comparisons between treatment-associated reductions in biomarker levels between the two study groups.** ^§^*P* = 0.037, ^§§^*P* = 0.011, compared with HΔ.

Comparative analysis of the changes in ICAM-1 (IACM-1Δ) and TNF-α (TNF-αΔ) levels between the H group and the HD groups revealed a more pronounced decrease in the HD group (ICAM-1Δ: 0.55 ± 0.64 pg/ml to 1.01 ± 0.80 pg/ml, *P* = 0.037; TNF-αΔ: 20.84 ± 14.89 pg/ml to 32.23 ± 14.33 pg/ml, *P* = 0.011, respectively, Figure 3).

### The Relation Between the Effect of Combined Treatment on Serum Biomarkers for Endothelial Function and Inflammation and BP and Cholesterol Levels

In bivariate correlation analysis, no significant correlations were apparent in either group between changes in ICAM-1 or TNF-α levels and changes in BP, Tc or LDL-c (Table 3).

**Table 3:** **Bivariate correlation analysis of treatment associated changes in serum intercellular adhesion molecule (ICAM)-1 and tumor necrosis factor (TNF)-α relative to those in blood pressure (BP), total cholesterol (Tc), and low density lipoprotein-cholesterol (LDL-c) levels**.

	SBPΔ		DBPΔ		TcΔ		LDL-cΔ	
	*r*	*P*	*r*	*P*	*r*	*P*	*r*	*P*
ICAM-1Δ	0.27	0.07	0.28	0.06	−0.24	0.12	−0.22	0.15
TNF-αΔ	0.28	0.07	0.08	0.59	0.15	0.32	0.17	0.28

*Δ denotes change in the corresponding indicator*.

## Discussion

The main findings of the present single center study were as follows: (1) patients with hypertension either with or without prediabetes appeared to benefit from treatment with single-pill combination of amlodipine 5 mg and atorvastatin calcium 10 mg, as reflected by significant reductions in ICAM-1 and TNF-α levels; and (2) said reductions in ICAM-1 and TNF-α levels were more pronounced in hypertensives with than without prediabetes, and independent of BP or cholesterol level reductions.

Our previous study had already shown that hypertensive prediabetics experienced unfavorable shifts toward higher levels of serum levels of ICAM-1 and TNF-α when compared with patients with isolated hypertension, suggesting a higher risk for CVD and warranting investigation of appropriate treatment interventions. The results of the present study are consistent with those of a study using an animal model, in which amlodipine plus atorvastatin significantly reduced TNF-α levels (Kawai et al., 2011; Zhang et al., 2012), and with the results of the MARGAUX study in which the said combination treatment significantly reduced the levels of adhesion molecules in human coronary disease (Charbonneau et al., 2008). The ASCOT-LLA study had shown important and large relative reductions in the rates of cardiovascular events associated with the additional use of atorvastatin 10 mg among hypertensive patients who were at moderate cardiovascular risk (hypertension with at least three cardiovascular risk factors; Sever et al., 2003). While the population in the present study was at lower risk (only age >45 years, hypertension or smoking as risk factors), short-term treatment of hypertensives with or without prediabetes with amlodipine plus atorvastatin calcium was enough to reduce ICAM-1 and TNF-α levels, with the effect being more pronounced in the presence of both conditions, indicating that use of this combination in such lower risk population can also improve endothelial function and inflammation. The latter anti-inflammatory effects appeared independent of the cholesterol and BP lowering effect of the combination treatment. Indeed, no significant correlation was found between the changes in TNF-α or ICAM-1 levels and the reduction in blood lipid levels of BP produced by the single-pill combination of amlodipine and atorvastatin calcium in the present study. The latter pleiotropic effects of amlodipine plus atorvastatin may contribute to the reported positive effect of statins (Schwartz et al., 2001; Fogari et al., 2006).

## Limitations

Interpretation of the results of the present study is limited by its single center, nonrandomized design with a relatively small sample size. Further larger, multicenter, randomized studies are warranted.

## Conclusion

In the present single center, non-randomized, small study, combined treatment with amlodipine and atorvastatin appeared to have a beneficial effect on endothelial function and inflammation, as reflected by reductions in serum ICAM-1 and TNF-α levels in hypertensives with or without prediabetes, with the effect being more pronounced in the former. Based on the latter findings, one could posit that starting statin treatment before overt diabetes becomes apparent in hypertensive patients with prediabetes might result in better cardiovascular outcomes, which needs to be tested in larger studies.

## Author Contributions

WH and ZH designed experiments; CC and FK carried out experiments; SL and PS analyzed experimental results. CC and ZH wrote the manuscript.

## Conflict of Interest Statement

The authors declare that the research was conducted in the absence of any commercial or financial relationships that could be construed as a potential conflict of interest. The reviewer TP and handling Editor declared their shared affiliation, and the handling Editor states that the process nevertheless met the standards of a fair and objective review.
